# Treatment of Active Crohn’s Disease in Children Using Partial Enteral Nutrition Combined with a Modified Crohn’s Disease Exclusion Diet: A Pilot Prospective Cohort Trial on Clinical and Endoscopic Outcomes

**DOI:** 10.3390/nu15214676

**Published:** 2023-11-04

**Authors:** Darja Urlep, Rok Orel, Patricija Kunstek, Evgen Benedik

**Affiliations:** 1 Department of Gastroenterology, Hepatology and Nutrition, University Children’s Hospital Ljubljana, 1000 Ljubljana, Slovenia; darja.urlep@gmail.com (D.U.); rok.orel@kclj.si (R.O.); patricija.kunstek@kclj.si (P.K.); 2 Biotechnical Faculty, University of Ljubljana, 1000 Ljubljana, Slovenia

**Keywords:** partial enteral nutrition, exclusive enteral nutrition, Crohn’s disease exclusion diet, paediatric patient

## Abstract

Background: Partial enteral nutrition (PEN) coupled with the Crohn’s disease (CD) exclusion diet (CDED) was shown to be effective in inducing clinical remission in paediatric CD. There are currently no robust data on the endoscopic outcomes of PEN. The aim of this study was to evaluate the clinical and endoscopic rates of remission after PEN combined with a modified CDED (mCDED) adjusted to the local cuisine in comparison with exclusive enteral nutrition (EEN) for the induction of remission. Methods: Between June 2017 and February 2021, a prospective cohort study on children with active CD, treated with PEN + mCDED or EEN, was performed at a single tertiary centre. Results: During the study period, 54 patients were screened and 15 were excluded according to the exclusion criteria, with six patients excluded in the first two days due to intolerance of the enteral formula. Fourteen patients were included in the PEN and 19 in the EEN group. They were assessed at Weeks 0, 1, 3 and 6, using clinical and laboratory parameters. Endoscopy was performed at Weeks 0 and 6. Clinical remission rates per protocol analysis were 84.6% in the PEN group and 81.3% in the EEN group (*p* = 0.99). At Week 6, an endoscopic response (a decline in the Simple Endoscopic Score for CD (SES-CD) > 50%) was observed in 84.6% of patients on PEN and in 68.8% on EEN treatment (*p* = 0.41). Endoscopic remission (SES-CD ≤ 2) was achieved in 53.8% of patients in the PEN group and in 50.0% in the EEN group (*p* = 0.99), while the mucosal healing rates (SES-CD = 0) were 38.5% with PEN and 43.8% with EEN (*p* = 0.99). A significant decline in the clinical and endoscopic activity scores was observed in both groups. Conclusion: Our study suggests that PEN + mCDED could be effective in inducing endoscopic remission and mucosal healing in active paediatric CD patients. Here, we present an analysis of the data from our cohort of patients and our real-world experience with PEN + mCDED.

## 1. Introduction

Crohn’s disease (CD) is a chronic immune-mediated disease that belongs to the group of inflammatory bowel diseases (IBD), along with ulcerative colitis (UC) and unclassified colitis. In Crohn’s disease, inflammation can occur anywhere throughout the gastrointestinal tract (GIT) from the mouth to the anus and can spread through the GIT mucosa to the deeper layers of the GIT wall, sometimes progressing through the bowel wall to the surrounding tissue, producing fistulas and abscesses [1].

Chronically active CD inflammation can lead to long-term complications such as stenotic and/or penetrating disease, stunted growth and developmental delay in children, and an increased incidence of intestinal neoplasia [1,2]. Early control of mucosal inflammation is therefore crucial in the treatment of CD [2,3].

Although the exact mechanisms of the pathogenesis of CD remain unknown, it has been hypothesised that complex interactions between genetic factors, the immune system, the microbiota, and environmental factors play a critical role in the development of the disease [4,5].

In the last few decades, the incidence of IBD has been steadily increasing in developed and developing parts of the world [6,7]. An increase in the incidence of IBD has also been widely reported for paediatric populations internationally [8]; the rise has been particularly noticeable in countries with previously historically low prevalence, including Slovenia [9,10,11]. The increase in the rate of IBD has been linked to the ever more prevalent Western lifestyle [12,13] and a dietary shift towards the Western diet, which is characterised by excessive consumption of saturated fats and sugars, and an insufficient intake of dietary fibre [14,15]. Dietary factors play a significant role in the pathogenesis of CD; however, their exact mechanisms of influence on intestinal inflammation are currently not yet clearly understood [16]. 

The efficacy of exclusive enteral nutrition (EEN) in inducing remission in active CD clearly demonstrates the significant impact of nutrition on intestinal inflammation in CD [17,18].

According to the European Society of Paediatric Gastroenterology, Hepatology and Nutrition (ESPGHAN) and the guidelines of the European Crohn’s and Colitis Organisation (ECCO), either EEN or corticosteroid (CS) therapy can be used for the induction of remission in active paediatric CD; however, EEN is used as a first-line therapy in mild and moderate non-penetrating paediatric CD [19,20]. In the case of fistulizing, penetrating disease, therapy with antibiotics and the early initiation of anti-tumour necrosis factor (anti-TNF) therapy is recommended [2,19]. For maintenance therapy, immunosuppressives and/or biological agents are used [2,19,20]. Compared with other treatments, EEN has numerous advantages and, most importantly, an absence of serious side effects [17,18,19,21,22,23].

Paediatric patients with newly diagnosed CD are often malnourished and have growth impairment at diagnosis. In this subgroup of patients, the initiation of CS therapy should be carefully considered due to its negative impact on growth [24,25].

Recent data have indicated that growth impairment presents an important predictive factor for later surgery in children and adolescents with CD [26]; therefore, timely escalation of the treatment to anti-TNF should be initiated to avoid serious complications [19]. 

In addition, several studies have confirmed that EEN is more effective than CS in achieving mucosal healing [27,28,29]. 

Despite its advantages, EEN is underused in clinical practice worldwide for the induction of remission in paediatric CD [30]. The most important cause preventing the widespread use of EEN is patient adherence. Adhering to the EEN regimen is difficult for most patients, as they are only allowed to consume a liquid enteral formula during the 6–8-week treatment period. Therefore, the concept of partial enteral nutrition (PEN) has recently come to the forefront of nutritional treatment research, as it allows patients to consume some solid food alongside the enteral formula [31,32,33,34,35] and helps to break the monotony of the EEN treatment. However, the first randomised controlled trial on PEN [35] reported low rates of clinical remission in children with active CD. In this study, 50% of dietary needs were covered by a liquid formula and 50% with an unrestricted diet [35]. Another two paediatric studies on PEN using an unrestricted diet also showed rates of clinical remission of 65% [36] and 50% [37]. Recently, several studies have reported higher rates of clinical remission when PEN was coupled with a special Crohn’s disease exclusion diet (CDED)—up to 75% [31,32,33,34,38,39]. These findings suggest that the CDED, which excludes potentially harmful dietary components that negatively affect intestinal permeability, the gut microbiome and gut immune mechanisms involved in the pathogenesis of CD, has a significant impact on gut inflammation [32,33,34,40,41]. The CDED was designed by Israeli authors [33,34], and its most important feature is the exclusion of processed foods with additives, animal fat, sugar, dairy products, and gluten [40,41]. 

However, all studies on PEN to date have lacked an endoscopic evaluation, including the only multicentric prospective randomised controlled trial (RCT) on PEN + CDED in children [34]. Achieving endoscopic remission is an important goal, both in research and real-world clinical practice [42,43,44]. Therefore, the primary aim of our study was to evaluate the clinical and endoscopic response, remission and mucosal healing rates after PEN combined with the modified CDED (mCDED) nutritional treatment. In our previously published pilot study, we reported that the PEN + mCDED treatment was effective in inducing an endoscopic response and remission [39] in children with active CD. In our clinical practice, this treatment approach has been in use since 2017. Here, we present further analysis of the data of a larger cohort of children with active CD and our real-world experience with PEN treatment combined with the mCDED.

## 2. Materials and Methods

### 2.1. Study Design and Patients

We conducted a prospective cohort study on children (aged ≤18) with active CD (newly diagnosed diseases and exacerbations) who were treated with nutritional therapy for the induction of remission at Ljubljana Children’s Hospital, University Centre Ljubljana, Slovenia, through the period from June 2017 to February 2021. All included patients were diagnosed according to the revised Porto criteria [45]. The inclusion criteria were clinically (Paediatric CD Activity Index (PCDAI) > 10) and endoscopically active CD defined by a Simple Endoscopic Score for CD (SES-CD) of >3. Exclusion criteria were PCDAI ≤ 10, SES-CD ≤ 3, penetrating disease (abscess or fistula), active perianal disease or extraintestinal disease, fixed strictures, obstruction of the small bowel, changes in maintenance treatment or having received steroids in the last 3 months prior to inclusion. All eligible patients were recruited into either the EEN or PEN nutritional group by their choice. The study was conceived as an RCT; however, most of the patients and their parents did not agree with randomisation because they wanted to choose their nutritional treatment regimen. 

### 2.2. Nutritional Therapy and Data Collection with Follow-Up

The first group of patients (PEN + mCDED) was treated according to our PEN + mCDED protocol [39]. Seventy-five percent of their daily dietary needs were covered by a polymeric formula (Alicalm, Nutricia, the Netherlands), and the remaining 25% by one permitted meal per day from the CDED, which was adapted to fit our Slovenian cuisine (mCDED). CDED and our mCDED are high-protein nutritionally balanced diets that exclude some potentially harmful dietary components such as food additives, sugar, animal fat, dairy products and gluten [32,33,34,40,41]. Recent studies in animal models and cell lines have pointed to the negative effect of food additives on the microbiome, intestinal permeability and gut immune mechanisms [32,33,34,40,41,46,47]. In the study by Chassaing et al., dietary emulsifiers (carboxymethylcellulose and polysorbate-80) induced low-grade inflammation of the colon in wild-type mice and produced robust inflammation in genetically susceptible mice [48]. Both the original CDED and our mCDED do not allow cow’s milk and dairy products. Milk-derived saturated fat was found to promote hepatic conjugation of bile acids with taurine and to increase the amount of luminal organic sulphur that leads to the expansion of the sulphite-reducing pathobiont *Bilophila wadsworthia*. This pathobiont was found to promote the Th-1 immune response in genetically susceptible mice, leading to the development of gut inflammation [49]. The exclusion of gluten from the original diet and our mCDED is based on a growing body of evidence showing that intestinal exposure to gliadin, a component of gluten, may lead to increased intestinal permeability [50,51,52,53]. In the study by Lammers et al., gliadin increased intestinal permeability by binding to the chemokine receptor CXCR3 and promoting the expression of zonulin in the gut’s lining cells [54].

Our mCDED differs slightly from the original, as it only allows patients to consume regionally grown fruits and vegetables, and locally and ecologically sourced white meat or fish. Among the carbohydrate-rich foods, buckwheat and millet were included alongside rice and potatoes, as they are part of traditional Slovenian cuisine (Table 1).

To make the PEN protocol simple and patient-friendly, the patients were encouraged to consume the same amount of food in each allowed meal as they were previously accustomed to. The second group of patients was treated with the standard EEN protocol which has been in use at our centre since 2002 (100% of daily energy needs covered by a polymeric formula plus an additional 20% of daily energy needs for catch-up growth) [39]. For the purposes of this study, the same polymeric formula (Alicalm, Nutricia, The Netherlands) was used for all enrolled patients.

The patients were followed by a paediatric gastroenterologist and a clinical and a research dietitian. They underwent physical examinations using the PCDAI, laboratory testing (erythrocyte sedimentation rate (ESR), C-reactive protein (CRP), haemoglobin, thrombocytes, serum albumin and faecal calprotectin (FC)) at baseline and after 1, 3, and 6 weeks of nutritional treatment. Ileocolonoscopies (complete colonoscopies with ileal intubation) were performed at baseline and at the end of the study period by trained paediatric endoscopists, who calculated the SES-CD score [55]. Before entering the patients into the study, we explained the need for a repeat ileocolonoscopy at the end of the EEN and PEN treatment. Patients were informed that their individual endoscopic response to the nutritional treatment was an important end goal, and that the endoscopic response often does not match clinical and laboratory improvements, including FC levels. Furthermore, evaluating their individual endoscopic response to the chosen enteral nutrition treatment was of further importance in the case of any future disease flares. At our centre, endoscopic procedures are performed under deep sedation and not under general anaesthesia; this may be the reason why endoscopic procedures are more acceptable to the patients.

Adherence to the nutritional protocol was assessed using a 24-h dietary recall questionnaire. The patients reported the amount and type of all foods and liquids as well as the volume of enteral formula consumed. Adherence was first assessed by the gastroenterologist at each visit. Twenty-four-hour recall was further analysed by the dietitian, who provided further feedback and gave dietary advice to the patients. The patients and their parents were encouraged to call at any time during the day when they had any trouble or questions regarding the treatment. With the intention to increase the adherence rate in our study, we tried to establish mutual trust among the patients, their parents, the gastroenterologist and the dietitian. We encouraged whole families to adhere to the mCDED and not buying sweets, processed food or snacks to avoid tempting their children. 

### 2.3. Flowchart of Patients throughout the Study

**Figure 1:**
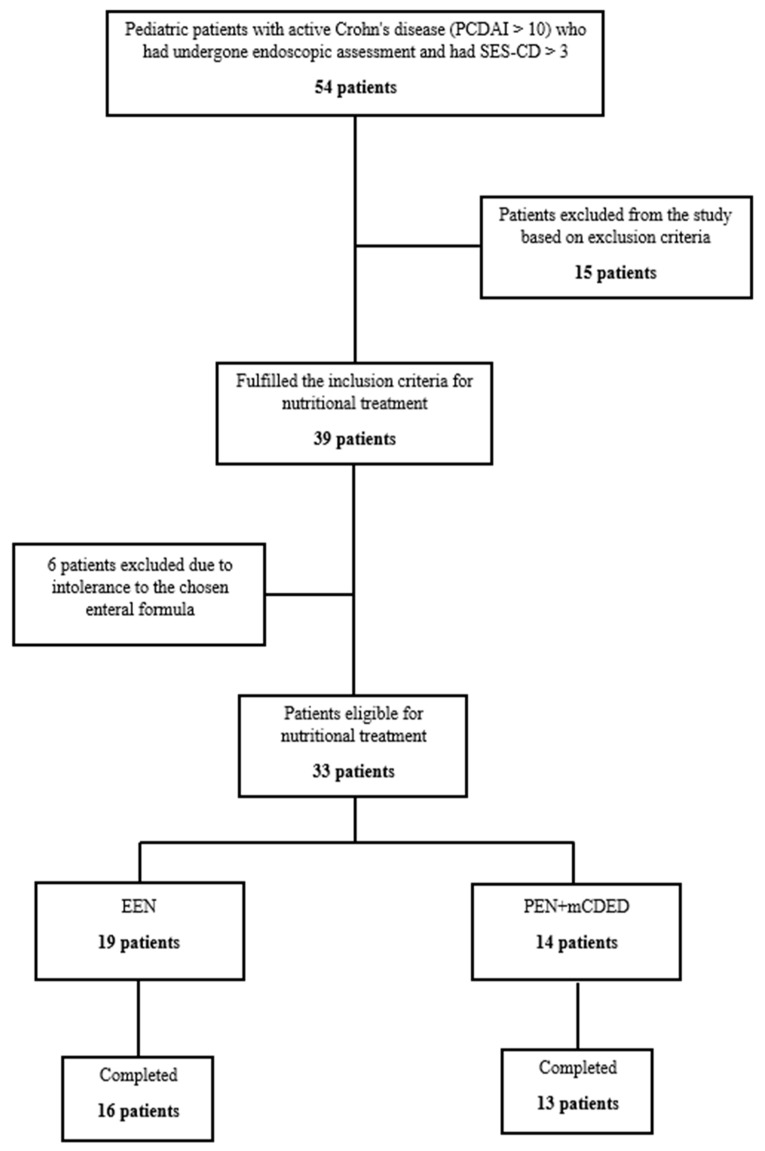
Flowchart of the patients throughout the study. Abbreviations: SES-CD, simple endoscopic score for Crohn’s disease; EEN, exclusive enteral nutrition; PEN + mCDED, partial enteral nutrition + modified Crohn’s disease exclusion diet.

In the study period from June 2017 until February 2021, 54 children with active CD (PCDAI > 10, SES-CD > 3) were screened for inclusion in the study. Figure 1 shows the progression of patients throughout the study. Fifteen patients were excluded according to the exclusion criteria. Thirty-nine patients were eligible for nutritional treatment; however, six patients were additionally excluded in the first 2 days as they did not tolerate the taste of the study’s enteral formula. Finally, 33 patients were included in the intention to treat (ITT) analysis, of whom 14 were included in the PEN and 19 in the EEN group. In the PEN group, one patient dropped out of the study within the first 4 days due to nausea and non-adherence; all others completed 6 weeks of the PEN treatment. In the EEN group, three patients dropped out in the first 4 days, of whom one did not tolerate the taste of the study formula, and two other patients suffered from vomiting and hematemesis. Both patients were found to have severe CD inflammation in the stomach with ulcers and epitheloid granulomas detected by pathohistology, and were later successfully treated with CS. Our study was active during the COVID-19 pandemic; 4 patients with an exacerbation of CD after a mild COVID-19 infection were excluded according to our study’s exclusion criteria. No patients tested positive at inclusion or at any of the follow-ups during nutritional therapy. 

### 2.4. Primary and Secondary Endpoints

The primary outcomes were clinical remission (PCDAI < 10), endoscopic remission (SES-CD ≤ 2) [56] and mucosal healing (a complete lack of endoscopically visible inflammation (SES-CD = 0) [56]. Secondary outcomes were the clinical response (a reduction in the PCDAI of ≥15), the endoscopic response (a decrease in SES-CD from the baseline of at least 50%), changes in mean values of PCDAI and SES-CD, changes in the laboratory data (ESR, CRP, haemoglobin, thrombocytes, serum albumin and FC) and the adherence rate.

### 2.5. Statistical Analysis

Baseline demographic and clinical data are presented as median with minimum and maximum values if numeric, and as frequencies and percentages if categorical. Comparisons of numeric variables between the two patient groups were performed using the Mann–Whitney U test, while the chi-square test or Fisher’s exact test was used for assessing differences in the distribution of categorical variables. A two-way repeated analysis of variance (ANOVA) was conducted to test whether there was a significant change in the outcome variables (PCDAI, SES-CD and the laboratory parameters) over the weeks of the treatment between the EEN and PEN patient groups. The main effects, i.e., the outcome measures and the weeks of treatment, and their interaction were included in the model. The results are presented as the estimated means with the standard errors. Statistical analysis was performed with SPSS 20 software (SPSS Inc., Chicago, IL, USA) and with the R language for statistical computing (R version 3.4.4). *p*-values less than 0.05 were considered to be statistically significant.

### 2.6. Ethical Issues

All participants received detailed information on the study and agreed with all procedures that were carried out. Written informed consent was obtained from all individual participants included in the study (from one of the parents in the case of children; in the case of adolescents, both from one parent and the participating adolescent). 

The study was approved by the Slovenian National Medical Ethics Committee (Ministry of Health, Ljubljana, Republic of Slovenia) in accordance with the Declaration of Helsinki (identification number: 0120-66/2016-2, KME 67/02/16). The trial was registered with ClinicalTrials.gov, NCT03176875.

## 3. Results

The baseline characteristics of the patients included in the study of the per-protocol analysis are presented in Table 2. Patients did not significantly differ between groups regarding important baseline characteristics, especially in the clinical and endoscopic activity of CD.

### 3.1. Clinical and Endoscopic Outcomes

**Figure 2:**
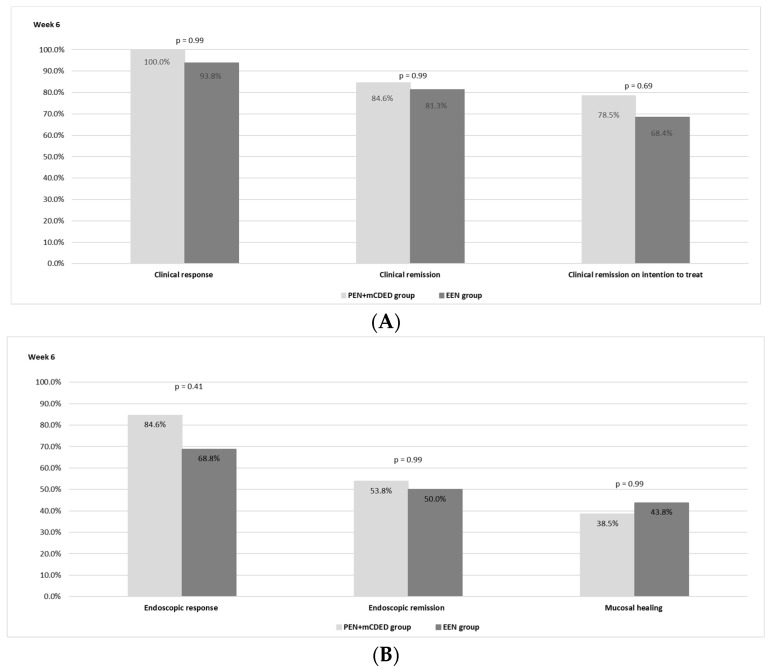
(**A**)The rates of clinical response and clinical remission in the per protocol and intention to treat analyses after 6 weeks of the PEN + mCDED and EEN treatment. (**B**) The rates of endoscopic response, endoscopic remission and mucosal healing after 6 weeks of the PEN + mCDED and EEN treatments. Abbreviations: PEN + mCDED, partial enteral nutrition + modified Crohn’s disease exclusion diet; EEN, exclusive enteral nutrition.

The clinical outcomes after 6 weeks of the nutritional treatment for both groups are shown in Figure 2A. There was no statistically significant difference observed between the PEN and the EEN groups regarding the rate of clinical remission. In the PEN + mCDED group, clinical remission (PCDAI < 10) on ITT analysis was achieved in 11/14 (78.5%) patients; in the EEN group, 13/19 (68.4%) patients achieved clinical remission (*p* = 0.69). Clinical remission in the per-protocol analysis was observed in 11/13 (84.6%) patients in the PEN + mCDED group and in 13/16 patients (81.3%) in the EEN group (*p* = 0.99). A clinical response was achieved in all patients (13/13) in the PEN + mCDED group and in 15 out of 16 (93.8%) in the EEN group.

Endoscopic remission (SES-CD ≤ 2) was achieved in 7/13 patients (53.8%) in the PEN + mCDED group and in 8/16 (50.0%) patients in the EEN group (*p* = 0.99), while mucosal healing rates (SES-CD = 0) were 38.5% with PEN + mCDED and 43.8% with EEN (*p* = 0.99). An endoscopic response (a decrease in the SES-CD score > 50% from the baseline) was observed in 11/13 (84.6%) patients in the PEN + mCDED group and in 11/16 (68.8%) patients in the EEN group (*p* = 0.41). The endoscopic outcomes are presented in Figure 2B.

### 3.2. Changes in the PCDAI and SES-CD Scores during Treatment with PEN + mCDED and EEN

**Figure 3:**
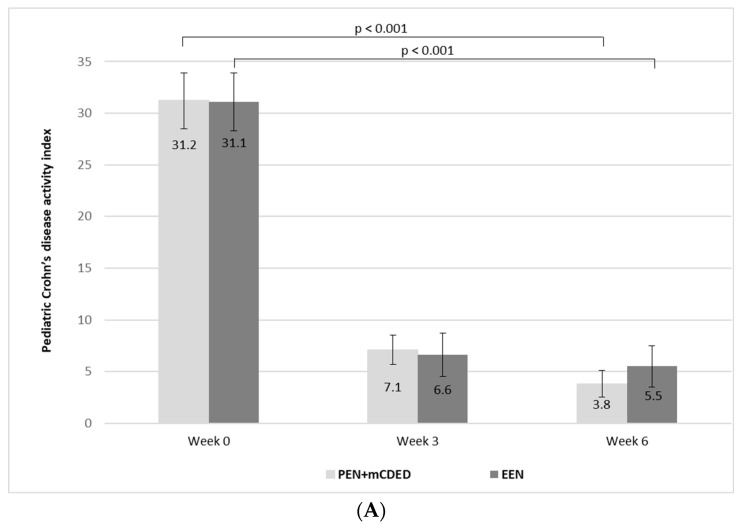

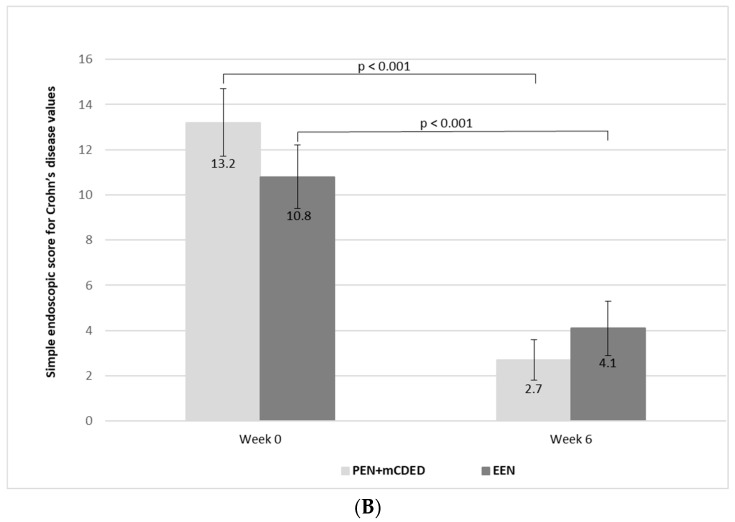
(**A**) Mean paediatric Crohn’s disease activity index values (with standard errors) at the baseline (Week 0), after 3 weeks and at the end of the treatment (Week 6) with the partial enteral nutrition + modified Crohn’s disease exclusion diet (PEN + mCDED) and exclusive enteral nutrition (EEN). (**B**) Mean simple endoscopic score for Crohn’s disease (with standard error) at the baseline (Week 0) and at the end of the treatment (Week 6) with the partial enteral nutrition + modified Crohn’s disease exclusion diet (PEN + mCDED) and exclusive enteral nutrition (EEN).

The mean values of PCDAI significantly decreased from the baseline to the end of the treatment in both groups (from 31.2 to 2.7 (standard error (SE)) to 7.1 (SE 1.4) at Week 3 and to 3.8 (SE 1.3) at Week 6 in the PEN + mCDED group (*p* < 0.001), and from 31.1 (SE 2.8) to 6.6 (SE 2.1) at Week 3 to 5.5 (SE 2.0) at Week 6 in the EEN group (*p* < 0.001). There was no significant difference in the decrease in mean PCDAI between the groups during the 6-week treatment (*p* = 0.88) (Figure 3A).

There was a significant decline in the mean values of the SES-CD score from the start to the end of the treatment in both groups (from 13.2 (SE 1.5) to 2.7 (SE 0.9) in the PEN + mCDED group (*p* < 0.001) and from 10.8 (SE 1.4) to 4.1 (SE 1.2) in the EEN group (*p* < 0.001)). There was no statistically significant difference in the decline in the SES-CD score between the two groups (*p* = 0.75) (Figure 3B).

### 3.3. Changes in the Laboratory Parameters during Treatment with PEN + mCDED and EEN

In both groups, the mean values of ESR, CRP, thrombocytes and FC decreased significantly from the baseline to the end of the treatment with PEN + mCDED (*p* = 0.001 for ESR, *p* = 0.044 for CRP, *p* = 0.006 for thrombocytes and *p* < 0.001 for FC) and with EEN (*p* = 0.003 for ESR, *p* = 0.006 for CRP, *p* = 0.004 for thrombocytes and *p* < 0.001 for FC).

The values of serum albumin significantly increased in both groups (*p* = 0.014 for the PEN + mCDED group and *p* = 0.012 for the EEN group); however, there was no statistically significant change in the mean values of haemoglobin in either group (*p* = 0.129 for the PEN + mCDED group and *p* = 0.208 for the EEN group). The changes in the laboratory parameters did not significantly differ between the two groups (Table 3).

### 3.4. Adherence

In terms of adherence, 13 out of 13 (100%) patients in the PEN + mCDED group who completed the 6 weeks of treatment adhered to the study protocol. Full adherence (16/16; 100%) was also observed at Week 6 in the EEN group.

## 4. Discussion

Evidence onthe endoscopic outcomes of the PEN + CDED treatment strategy is currently lacking, even though endoscopic evaluation is now considered a key primary endpoint, both in clinical trials and in real-life practice [42,43,44,58]. Our study is the first that evaluated the endoscopic outcomes after the induction of remission with the PEN treatment in children with active CD.

In our study, the rate of clinical remission on per-protocol analysis after 6 weeks of treatment in the PEN + mCDED group (84.6%) did not significantly differ from that observed in the EEN group (81.3%). Our rate of clinical remission in the PEN + mCDED group is among the highest reported for PEN + CDED in the literature [31,32,33,34,38] and is similar to the observed rates published for EEN [17,18,19,20,21,22,23,24]. The rate of clinical remission in the ITT analysis in our PEN + mCDED group (78.5%) is in accordance with the rate that was reported in the RCT by Israeli and Canadian authors (75%) [34]. In this only paediatric prospective RCT to date on PEN, this treatment strategy (50% PEN + CDED) was shown to be as effective as EEN in inducing clinical remission in the ITT analysis (75% vs. 59%; *p* = 0.14) after 6 weeks of treatment. This study consisted of an additional 6-week period following the first one, where the PEN group received 25% PEN combined with CDED and the control group received 25% PEN with an unrestricted diet. The PEN + CDED group achieved better remission rates at Week 12 [34]. The effectiveness of PEN + CDED for the induction of remission is also supported by recent real-world retrospective paediatric studies [31,38,59,60]. Surprisingly, in the recently published adult multicentric RCT, high PEN + CDED efficacy was observed even in adult CD patients [61]. PEN + CDED has also been found to be an effective treatment strategy in CD patients failing biologic therapy [38,62].

In our study, the rates of endoscopic response, remission and mucosal healing were 84.6%, 53.8% and 38.5%, respectively, after only 6 weeks of treatment with PEN + mCDED. Because our study is the first that evaluated the endoscopic outcomes of the PEN treatment for the induction of remission, we can only compare our results with those reported for EEN therapy. Between our PEN + mCDED and EEN groups, no statistically significant difference was found in the rates of endoscopic response, remission and mucosal healing (68.8%, 50.0%, and 43.8% in the EEN group, respectively), although the sample sizes were small. In an RCT by Borrelli et al., similarly high endoscopic remission rates (14/19; 74%) were observed in children with active CD after 10 weeks of the EEN treatment [29]; however, they defined endoscopic remission less rigorously (as a decrease in the endoscopic and histologic scores by 50%) and the endoscopic assessments were performed after a longer period of nutritional treatment [29]. In a study by Canani et al., complete mucosal healing was achieved in a lower percentage of children (27%) with active CD after 8 weeks of EEN [28], compared with the 6-week treatment period in our PEN + mCDED and EEN groups. A slightly lower rate of mucosal healing was observed in a prospective study by Grover et al., where SES-CD scores were used as well [27]. In their study, mucosal healing (defined as SES-CD = 0) was observed in 33% of patients who completed 6 weeks of treatment with EEN. However, our rates of endoscopic remission cannot be directly compared with those reported by Grover et al., [27] as they did not define the endoscopic outcomes strictly according to the recent endoscopic recommendations in children with IBD [56]. Therefore, larger RCTs on PEN evaluating the endoscopic outcomes based on the current endoscopic guidelines are needed.

A significant decline in the mean values of PCDAI, the SES-CD score, ESR, CRP and the number of thrombocytes together with the significant increase in the mean serum albumin values in the PEN + mCDED and EEN groups additionally support the finding of clinical and endoscopic improvement after a 6-week period of either nutritional treatment strategy. A rapid decline in the mean values of PCDAI (from 31.2 to 7.1 with PEN + mCDED and from 31.1 to 6.6 with EEN) after 3 weeks of treatment was observed. This is consistent with the recent findings of a study by Sigall-Boneh et al. [32], indicating the rapid efficiency of PEN + CDED. It is worth noting that there was no significant change in haemoglobin levels in either of the treatment groups. This finding is in line with the results of a recent paediatric real-world study from Spain, where an increase in haemoglobin levels was found only after 24 weeks of PEN treatment [38]. In light of this finding, appropriate management of anaemia during nutritional treatment should be emphasised [63].

All our patients who completed the PEN + mCDED and EEN treatment protocols claimed 100% adherence to the study protocol. We can speculate that the high clinical and endoscopic remission rates after only 6 weeks of nutritional treatment may be associated with high adherence in both groups of our patients. Indeed, adherence has been reported to have a crucial impact on the response to EEN and PEN therapy [38,64,65,66]. In a New Zealand study, parental support and engagement, and the support of dietitians and other medical professionals were among the most important factors affecting the rate of adherence to EEN in paediatric CD [67].

We acknowledge several limitations of this study. The main one is the small sample size of each treatment group. Our study is not sufficiently powered to reliably detect differences between the outcomes of the two nutritional treatment strategies, as the differences in the rates of remission between PEN + CDED and EEN reported in the literature are already small. Another limitation of this study is that the patients were not randomised into the two nutritional treatment groups. When we informed our patients about the randomised study protocol, most of them did not agree to be included in the study. They wanted to choose their own type of nutritional treatment. Interestingly, almost all children and adolescents chose PEN + mCDED over EEN. However, some of their parents chose EEN, as it is the only currently officially recommended nutritional treatment [19,68]. We can speculate that the high adherence rate in our study was the consequence of the non-randomised study protocol. We believe that randomisation would lower the adherence to non-chosen nutritional treatments, reducing the overall effectiveness. It is worth noting that the higher proportion of PEN in our study (75%) may have also contributed to better clinical outcomes when compared with those reported in the literature, where only 50% PEN + CDED was used [31,32,33,34,59]. Further larger studies are warranted to find out the most effective ratio of PEN in the induction of remission. In a recent real-world study from Croatia, 80% of the patients who were treated with 50% PEN + CDED first underwent 1–2 weeks of EEN treatment prior to initiating PEN [31]. This strategy could be beneficial, especially in CD patients presenting with more active CD. We use a 75% PEN treatment approach in our clinical practice, since it allows the patients to enjoy one meal per day together with the whole family. We believe that this approach encourages the whole family to remain involved in the child’s nutritional treatment.

## 5. Conclusions

There is growing interest in the treatment of active Crohn’s disease using partial enteral nutrition in combination with the Crohn’s disease exclusion diet. However, no studies to date have included an endoscopic evaluation as part of their study design, even though endoscopic remission and mucosal healing are now considered key primary endpoints in IBD.

While taking note of the limitations of this study, especially the small sample size, our findings suggest that partial enteral nutrition coupled with the Crohn’s disease exclusion diet, modified to fit the local cuisine, may be as effective as exclusive enteral nutrition for the induction of remission in paediatric Crohn’s disease. However, larger controlled randomised studies on the clinical and endoscopic outcomes of partial enteral nutrition in combination with the Crohn’s disease exclusion diet are needed.

## Figures and Tables

**Table 1:** Modified Crohn’s disease exclusion diet (mCDED) based on traditional Slovenian cuisine.

**Excluded foods**
Fried foods
Processed foods and products with additives (emulsifiers, preservatives, maltodextrins and carrageenan) Processed or smoked meat and fish products (sausages, salami, smoked meat or fish, meat or fish paté) All canned dishes Sauces, salad dressings, mayonnaise, puddings, jams, and syrups Baked goods (breads, croissants, and doughnuts) Breakfast cereals Snacks (chips, dried fruit, nuts, pretzels, and popcorn) Packaged mixtures of spices with additives, prepackaged soup stock
Sugar and sweets (chocolates, candies, cookies, and cakes)
Red meat and animal fat
Cow’s milk and dairy products
Gluten-containing foods
Exotic fruit (such as citrus)
Cabbage, turnips, legumes (beans, peas, lentils and chickpeas)
Beverages with added sugar or alcohol of any kind
**Allowed foods**
Food may be boiled, broiled, baked or grilled
Ecologically and locally sourced fresh white meat and fish: Chicken, turkey and rabbit meat Fresh fish Organically sourced eggs (1–2 per day)
A small amount of honey
Unprocessed gluten-free food without yeast
Buckwheat, millet (encouraged), rice, cooked potatoes
Regionally grown fruit First 3 weeks, only boiled fruit; later, peeled fruit: apples, plums, pears, blueberries, raspberries, strawberries, apricots, cherries, grapes without peels and stones
Regionally grown vegetables Boiled vegetables: carrot, celery, cauliflower, broccoli, spinach, pumpkin, beet, leek, asparagus, brussels sprouts, German turnip, peeled and cooked tomato and cucumber Fresh vegetables: soft varieties of lettuce
Olive oil, canola oil, linseed oil, coconut oil and walnut oil
Condiments: salt, cooked or sautéed onion and garlic Fresh herbs: parsley, green, basil, oregano, coriander, sage, thyme and rosemary
Drinks: only water, mineral water, and unsweetened herbal tea

**Table 2:** Baseline characteristics of patients treated with partial enteral nutrition + modified Crohn’s disease exclusion diet (PEN + mCDED) and exclusive enteral (EEN) nutrition.

	PEN + mCDED (*n* = 13)	EEN (*n* = 16)	*p*
Age at inclusion (median, IQR)	14.0 (4.5)	14.1 (3.8)	0.726
Male, *n* (%)	8 (61.5)	6 (37.5)	0.198
Newly diagnosed, *n* (%)	6 (46.1)	12 (75.0)	0.143
Paris classification [57] Age at diagnosis *n* (%)			0.455
A1a (<10 years)	3 (23.1)	2 (12.5)	-
A1b (10–17 years)	10 (76.9)	12 (75.0)	-
A2 (>17 years)	0 (0)	2 (12.5)	-
Location, *n* (%)			0.948
L1—ileal/ileocecal	1 (7.7)	0 (0)	-
L2—colonic	4 (30.7)	7 (43.8)	-
L3—ileocolonic	8 (61.6)	9 (56.2)	-
L4a	11 (84.6)	12 (75.0)	-
L4b	3 (23.1)	2 (12.5)	-
L4ab—L4a + L4b	3 (23,1)	2 (12.5)	-
Duration of nutritional treatment (days)	47.5 (5.5)	49.3 (8.1)	0.505
Maintenance therapy at baseline, *n* (%)			
IMM only	5 (38.5)	4 (25.0)	
Azathioprine + anti-TNF	1 (7.7)	0	
Vedolizumab	1 (7.7)	0	
ESR	38.3 (7.0)	35.8 (5.6)	0.544
CRP	23.0 (6.6)	17.2 (2.9)	0.566
Baseline PCDAI score	31.2 (2.7)	31.1 (2.8)	0.544
Baseline SES-CD score	13.2 (1.5)	10.8 (1.4)	0.247

Abbreviations: PEN + mCDED, partial enteral nutrition + modified Crohn’s disease exclusion diet; EEN, exclusive enteral nutrition; IQR, interquartile range; IMM, immunomodulator; anti-TNF, anti-tumour necrosis factor; ESR, erythrocyte sedimentation rate; CRP, C-reactive protein; PCDAI, paediatric Crohn’s disease activity index; SES-CD, simple endoscopic score for Crohn’s disease. ESR, CRP, PCDAI, and SES-CD are presented as the mean and standard error.

**Table 3:** Changes in the mean values (with standard error) of the laboratory parameters during 6 weeks of treatment with partial enteral nutrition + the modified Crohn’s disease exclusion diet (PEN + mCDED) and exclusive enteral nutrition (EEN).

	PEN + mCDED					EEN					PEN + mCDED vs. EEN
Laboratory Parameters	Week 0	Week 1 ^a^	Week 3	Week 6	*p*	Week 0	Week 1 ^a^	Week 3	Week 6	*p*	*p*
ESR, mm/h	38.3 (7.0)	25.4 (4.6)	17.5 (2.6)	13.7 (1.3)	0.001	35.8 (5.6)	24.3 (3.7)	17.5 (3.4)	15.2 (3.1)	0.003	0.914
CRP, mg/L ^b^	23.0 (6.6)	8.7 (0.8)	8.0 (0)	7.9 (0)	0.044	17.2 (2.9)	10.0 (1.2)	8.9 (1.0)	7.9 (1.2)	0.006	0.869
Hemoglobin, g/L	121.6 (2.9)	124.8 (2.4)	126.3 (2.0)	123.3 (2.1)	0.129	118.2 (2.9)	119.0 (3.9)	118.2 (3.5)	114.7 (4.2)	0.208	0.100
Thrombocytes, ×10^9^/L	447.7 (40.4)	427.8 (32.3)	387.3 (27.1)	367.9 (30.0)	0.006	439.8 (29.2)	409.9 (28.7)	388.0 (28.6)	343.2 (25.7)	0.004	0.752
Albumin, g/L	39.3 (1.2)	41.5 (1.1)	42.3 (1.1)	42.9 (1.2)	0.014	38.2 (1.8)	39.9 (1.8)	41.5 (1.7)	41.7 (1.8)	0.012	0.536
FC, mg/kg ^c^	420.4 (29.9)	-	-	133.2 (20.6)	<0.001	380.1 (34.6)	-	-	171.4 (30.9)	<0.001	0.813

Abbreviations: PEN + mCDED, partial enteral nutrition + modified Crohn’s disease exclusion diet; EEN, exclusive enteral nutrition; ESR, erythrocyte sedimentation rate; CRP, C-reactive protein; FC, faecal calprotectin. ^a^ At Week 1, there were missing laboratory data in the PEN + mCDED group from one patient; in the EEN group, there were missing data from three patients. ^b^ Negative values of CRP < 8 are marked as 7.9. ^c^ At Week 6, there is one missing value for FC in the PEN + mCDED group and two missing values for FC in the EEN group.

## Data Availability

The datasets generated during and/or analysed during the current study are available from the corresponding author on reasonable request.

## References

[1] Torres J., Mehandru S., Colombel J.F., Peyrin-Biroulet L. (2017). Crohn’s disease. Lancet.

[2] Mitchel E.B., Rosh J.R. (2022). Pediatric Management of Crohn’s Disease. Gastroenterol. Clin. N. Am..

[3] Cushing K., Higgins P.D.R. (2021). Management of Crohn Disease: A Review. JAMA.

[4] Graham D.B., Xavier R.J. (2020). Pathway paradigms revealed from the genetics of inflammatory bowel disease. Nature.

[5] Cohen L.J., Cho J.H., Gevers D., Chu H. (2019). Genetic Factors and the Intestinal Microbiome Guide Development of Microbe-Based Therapies for Inflammatory Bowel Diseases. Gastroenterology.

[6] Ng S.C., Shi H.Y., Hamidi N., Underwood F.E., Tang W., Benchimol E.I., Panaccione R., Ghosh S., Wu J.C.Y., Chan F.K.L. (2017). Worldwide incidence and prevalence of inflammatory bowel disease in the 21st century: A systematic review of population-based studies. Lancet.

[7] Ananthakrishnan A.N., Kaplan G.G., Ng S.C. (2020). Changing Global Epidemiology of Inflammatory Bowel Diseases: Sustaining Health Care Delivery Into the 21st Century. Clin. Gastroenterol. Hepatol..

[8] Kuenzig M.E., Fung S.G., Marderfeld L., Mak J.W.Y., Kaplan G.G., Ng S.C., Wilson D.C., Cameron F., Henderson P., Kotze P.G. (2022). Twenty-first Century Trends in the Global Epidemiology of Pediatric-Onset Inflammatory Bowel Disease: Systematic Review. Gastroenterology.

[9] Urlep D., Blagus R., Orel R. (2015). Incidence Trends and Geographical Variability of Pediatric Inflammatory Bowel Disease in Slovenia: A Nationwide Study. Biomed. Res. Int..

[10] Urlep D., Trop T.K., Blagus R., Orel R. (2014). Incidence and phenotypic characteristics of pediatric IBD in northeastern Slovenia, 2002–2010. J. Pediatr. Gastroenterol. Nutr..

[11] Orel R., Kamhi T., Vidmar G., Mamula P. (2009). Epidemiology of pediatric chronic inflammatory bowel disease in central and western Slovenia, 1994-2005. J. Pediatr. Gastroenterol. Nutr..

[12] Rogler G., Vavricka S. (2015). Exposome in IBD: Recent insights in environmental factors that influence the onset and course of IBD. Inflamm. Bowel Dis..

[13] Bernstein C.N. (2017). Review article: Changes in the epidemiology of inflammatory bowel disease—Clues for aetiology. Aliment. Pharmacol. Ther..

[14] Hou J.K., Abraham B., El-Serag H. (2011). Dietary intake and risk of developing inflammatory bowel disease: A systematic review of the literature. Am. J. Gastroenterol..

[15] Adolph T.E., Zhang J. (2022). Diet fuelling inflammatory bowel diseases: Preclinical and clinical concepts. Gut.

[16] Schreiner P., Martinho-Grueber M., Studerus D., Vavricka S.R., Tilg H., Biedermann L. (2020). Nutrition in Inflammatory Bowel Disease. Digestion.

[17] Narula N., Dhillon A., Zhang D., Sherlock M.E., Tondeur M., Zachos M. (2018). Enteral nutritional therapy for induction of remission in Crohn’s disease. Cochrane Database Syst. Rev..

[18] Swaminath A., Feathers A., Ananthakrishnan A.N., Falzon L., Li Ferry S. (2017). Systematic review with meta-analysis: Enteral nutrition therapy for the induction of remission in paediatric Crohn’s disease. Aliment. Pharmacol. Ther..

[19] Van Rheenen P.F., Aloi M., Assa A., Bronsky J., Escher J.C., Fagerberg U.L., Gasparetto M., Gerasimidis K., Griffiths A., Henderson P. (2020). The Medical Management of Paediatric Crohn’s Disease: An ECCO-ESPGHAN Guideline Update. J. Crohns Colitis.

[20] Ruemmele F.M., Veres G., Kolho K.L., Griffiths A., Levine A., Escher J.C., Amil Dias J., Barabino A., Braegger C.P., Bronsky J. (2014). Consensus guidelines of ECCO/ESPGHAN on the medical management of pediatric Crohn’s disease. J. Crohns Colitis.

[21] Cohen-Dolev N., Sladek M., Hussey S., Turner D., Veres G., Koletzko S., Martin de Carpi J., Staiano A., Shaoul R., Lionetti P. (2018). Differences in Outcomes Over Time with Exclusive Enteral Nutrition Compared With Steroids in Children with Mild to Moderate Crohn’s Disease: Results From the GROWTH CD Study. J. Crohns Colitis.

[22] Van Limbergen J.E., Koot B.G.P., de Winter J.P. (2020). Fool me once… treatment exposure to achieve remission in pediatric IBD. Eur. J. Pediatr..

[23] Yu Y., Chen K.C., Chen J. (2019). Exclusive enteral nutrition versus corticosteroids for treatment of pediatric Crohn’s disease: A meta-analysis. World J. Pediatr..

[24] Ley D., Duhamel A., Behal H., Vasseur F., Sarter H., Michaud. L., Gower-Rousseau C., Turck D. (2016). Growth Pattern in Paediatric Crohn Disease Is Related to Inflammatory Status. J. Pediatr. Gastroenterol. Nutr..

[25] Shamir R., Phillip M., Levine A. (2007). Growth retardation in pediatric Crohn’s disease: Pathogenesis and interventions. Inflamm. Bowel Dis..

[26] Ricciuto A., Aardoom M., Orlanski-Meyer E., Navon D., Carman N., Aloi M., Bronsky J., Däbritz J., Dubinsky M., Hussey S. (2021). Predicting Outcomes in Pediatric Crohn’s Disease for Management Optimization: Systematic Review and Consensus Statements from the Pediatric Inflammatory Bowel Disease-Ahead Program. Gastroenterology.

[27] Grover Z., Muir R., Lewindon P. (2014). Exclusive enteral nutrition induces early clinical, mucosal and transmural remission in paediatric Crohn’s disease. J. Gastroenterol..

[28] Berni Canani R., Terrin G., Borrelli O., Romano M.T., Manguso F., Coruzzo A., D’Armiento F., Romeo E.F., Cucchiara S. (2006). Short- and long-term therapeutic efficacy of nutritional therapy and corticosteroids in paediatric Crohn’s disease. Dig. Liver Dis..

[29] Borrelli O., Cordischi L., Cirulli M., Paganelli M., Labalestra V., Uccini S., Russo P.M., Cucchiara S. (2006). Polymeric diet alone versus corticosteroids in the treatment of active pediatric Crohn’s disease: A randomized controlled open-label trial. Clin. Gastroenterol. Hepatol..

[30] Van Limbergen J., Haskett J., Griffiths A.M., Critch J., Huynh H., Ahmed N., deBruyn J.C., Issenman R., El-Matary W., Walters T.D. (2015). Toward enteral nutrition for the treatment of pediatric Crohn disease in Canada: A workshop to identify barriers and enablers. Can. J. Gastroenterol. Hepatol..

[31] Niseteo T., Sila S., Trivić I., Mišak Z., Kolaček S., Hojsak I. (2022). Modified Crohn’s disease exclusion diet is equally effective as exclusive enteral nutrition: Real-world data. Nutr. Clin. Pract..

[32] Sigall-Boneh R., Van Limbergen J., Wine E., Assa A., Shaoul R., Milman P., Cohen S., Kori M., Peleg S., On A. (2021). Dietary Therapies Induce Rapid Response and Remission in Pediatric Patients with Active Crohn’s Disease. Clin. Gastroenterol. Hepatol..

[33] Sigall-Boneh R., Pfeffer-Gik T., Segal I., Zangen T., Boaz M., Levine A. (2014). Partial enteral nutrition with a Crohn’s disease exclusion diet is effective for induction of remission in children and young adults with Crohn’s disease. Inflamm. Bowel Dis..

[34] Levine A., Wine E., Assa A., Sigall Boneh R., Shaoul R., Kori M., Cohen S., Peleg S., Shamaly H., On A. (2019). Crohn’s Disease Exclusion Diet Plus Partial Enteral Nutrition Induces Sustained Remission in a Randomized Controlled Trial. Gastroenterology.

[35] Johnson T., Macdonald S., Hill S.M., Thomas A., Murphy M.S. (2006). Treatment of active Crohn’s disease in children using partial enteral nutrition with liquid formula: A randomised controlled trial. Gut.

[36] Gupta K., Noble A., Kachelries K.E., Albenberg L., Kelsen J.R., Grossman A.B., Baldassano R.N. (2013). A novel enteral nutrition protocol for the treatment of pediatric Crohn’s disease. Inflamm. Bowel Dis..

[37] Lee D., Baldassano R.N., Otley A.R., Albenberg L., Griffiths A.M., Compher C., Chen E.Z., Li H., Gilroy E., Nessel L. (2015). Comparative Effectiveness of Nutritional and Biological Therapy in North American Children with Active Crohn’s Disease. Inflamm. Bowel Dis..

[38] Jijón Andrade M.C., Pujol Muncunill G., Lozano Ruf A., Álvarez Carnero L., Vila Miravet V., García Arenas D., Egea Castillo N., Martín de Carpi J. (2023). Efficacy of Crohn’s disease exclusion diet in treatment -naïve children and children progressed on biological therapy: A retrospective chart review. BMC Gastroenterol..

[39] Urlep D., Benedik E., Brecelj J., Orel R. (2020). Partial enteral nutrition induces clinical and endoscopic remission in active pediatric Crohn’s disease: Results of a prospective cohort study. Eur. J. Pediatr..

[40] Pfeffer-Gik T., Levine A. (2014). Dietary clues to the pathogenesis of Crohn’s disease. Dig. Dis..

[41] Levine A., Sigall Boneh R., Wine E. (2018). Evolving role of diet in the pathogenesis and treatment of inflammatory bowel diseases. Gut.

[42] Sabino J., Lewis J.D., Colombel J.F. (2019). Treating Inflammatory Bowel Disease with Diet: A Taste Test. Gastroenterology.

[43] Pineton de Chambrun G., Blanc P., Peyrin-Biroulet L. (2016). Current evidence supporting mucosal healing and deep remission as important treatment goals for inflammatory bowel disease. Expert Rev. Gastroenterol. Hepatol..

[44] Baert F., Moortgat L., Van Assche G., Caenepeel P., Vergauwe P., De Vos M., Stokkers P., Hommes D., Rutgeerts P., Vermeire S. (2010). Mucosal healing predicts sustained clinical remission in patients with early-stage Crohn’s disease. Gastroenterology.

[45] Levine A., Koletzko S., Turner D., Escher J.C., Cucchiara S., De Ridder L., Kolho K.L., Veres G., Russell R.K., Paerregaard A. (2014). ESPGHAN revised porto criteria for the diagnosis of inflammatory bowel disease in children and adolescents. J. Pediatr. Gastroenterol. Nutr..

[46] Sasson A.N., Ananthakrishnan A.N., Raman M. (2021). Diet in Treatment of Inflammatory Bowel Diseases. Clin. Gastroenterol. Hepatol..

[47] Wellens J., Vissers E., Matthys C., Vermeire S., Sabino J. (2023). Personalized Dietary Regimens for Inflammatory Bowel Disease: Current Knowledge and Future Perspectives. Pharmgenomics Pers. Med..

[48] Chassaing B., Koren O., Goodrich J.K., Poole A.C., Srinivasan S., Ley R.E., Gewirtz A.T. (2015). Dietary emulsifiers impact the mouse gut microbiota promoting colitis and metabolic syndrome. Nature.

[49] Devkota S., Wang Y., Musch M.W., Leone V., Fehlner-Peach H., Nadimpalli A., Antonopoulos D.A., Jabri B., Chang E.B. (2012). Dietary-fat-induced taurocholic acid promotes pathobiont expansion and colitis in Il10-/- mice. Nature.

[50] Hollon J., Puppa E.L., Greenwald B., Goldberg E., Guerrerio A., Fasano A. (2015). Effect of gliadin on permeability of intestinal biopsy explants from celiac disease patients and patients with non-celiac gluten sensitivity. Nutrients.

[51] Drago S., El Asmar R., Di Pierro M., Clemente M.G., Tripathi A., Sapone A., Thakar M., Iacono G., Carroccio A., D’Agate C. (2006). Gliadin, zonulin and gut permeability: Effects on celiac and non-celiac intestinal mucosa and intestinal cell lines. Scand. J. Gastroenterol..

[52] Wagner S.J., Schmidt A., Effenberger M.J.P., Gruber L., Danier J., Haller D. (2013). Semisynthetic diet ameliorates Crohn’s disease-like ileitis in TNFΔARE/WT mice through antigen-independent mechanisms of gluten. Inflamm. Bowel Dis..

[53] Shimada S., Tanigawa T., Watanabe T., Nakata A., Sugimura N., Itani S., Higashimori A., Nadatani Y., Otani K., Taira K. (2019). Involvement of gliadin, a component of wheat gluten, in increased intestinal permeability leading to non-steroidal anti-inflammatory drug-induced small-intestinal damage. PLoS ONE.

[54] Lammers K.M., Lu R., Brownley J., Lu B., Gerard C., Thomas K., Rallabhandi P., Shea-Donohue T., Tamiz A., Alkan S. (2008). Gliadin induces an increase in intestinal permeability and zonulin release by binding to the chemokine receptor CXCR3. Gastroenterology.

[55] Daperno M., D’Haens G., Van Assche G., Baert F., Bulois P., Maunoury V., Sostegni R., Rocca R., Pera A., Gevers A. (2004). Development and validation of a new, simplified endoscopic activity score for Crohn’s disease: The SES-CD. Gastrointest. Endosc..

[56] Oliva S., Thomson M., De Ridder L., Martín-De-Carpi J., Van Biervliet S., Braegger C., Dias J.A., Kolacek S., Miele E., Buderus S. (2018). Endoscopy in Pediatric Inflammatory Bowel Disease: A Position Paper on Behalf of the Porto IBD Group of the European Society for Pediatric Gastroenterology, Hepatology and Nutrition. J. Pediatr. Gastroenterol. Nutr..

[57] Levine A., Griffiths A., Markowitz J., Wilson D.C., Turner D., Russell R.K., Fell J., Ruemmele F.M., Walters T., Sherlock M. (2011). Pediatric modification of the Montreal classification for inflammatory bowel disease: The Paris classification. Inflamm. Bowel Dis..

[58] Peyrin-Biroulet L., Sandborn W., Sands B.E., Reinisch W., Bemelman W., Bryant R.V., D’Haens G., Dotan I., Dubinsky M., Feagan B. (2015). Selecting Therapeutic Targets in Inflammatory Bowel Disease (STRIDE): Determining Therapeutic Goals for Treat-to-Target. Am. J. Gastroenterol..

[59] Matuszczyk M., Meglicka M., Wiernicka A., Jarzębicka D., Osiecki M., Kotkowicz-Szczur M., Kierkuś J. (2022). Effect of the Crohn’s Disease Exclusion Diet (CDED) on the Fecal Calprotectin Level in Children with Active Crohn’s Disease. J. Clin. Med..

[60] Scarallo L., Banci E., Pierattini V., Lionetti P. (2021). Crohn’s disease exclusion diet in children with Crohn’s disease: A case series. Curr. Med. Res. Opin..

[61] Yanai H., Levine A., Hirsch A., Boneh R.S., Kopylov U., Eran H.B., Cohen N.A., Ron Y., Goren I., Leibovitzh H. (2022). The Crohn’s disease exclusion diet for induction and maintenance of remission in adults with mild-to-moderate Crohn’s disease (CDED-AD): An open-label, pilot, randomised trial. Lancet Gastroenterol. Hepatol..

[62] Boneh R.S., Shabat C.S., Yanai H., Chermesh I., Ben Avraham S., Boaz M., Levine A. (2017). Dietary Therapy with the Crohn’s Disease Exclusion Diet is a Successful Strategy for Induction of Remission in Children and Adults Failing Biological Therapy. J. Crohns Colitis.

[63] Dignass A.U., Gasche C., Bettenworth D., Birgegård G., Danese S., Gisbert J.P., Gomollon F., Iqbal T., Katsanos K., Koutroubakis I. (2015). European consensus on the diagnosis and management of iron deficiency and anaemia in inflammatory bowel diseases. J. Crohns Colitis..

[64] Day A.S., Lopez R.N. (2015). Exclusive enteral nutrition in children with Crohn’s disease. World J. Gastroenterol..

[65] Critch J., Day A.S., Otley A., King-Moore C., Teitelbaum J.E., Shashidhar H. (2012). Use of enteral nutrition for the control of intestinal inflammation in pediatric Crohn disease. J. Pediatr. Gastroenterol. Nutr..

[66] Ashton J.J., Gavin J., Beattie R.M. (2019). Exclusive enteral nutrition in Crohn’s disease: Evidence and practicalities. Clin. Nutr..

[67] Ho S.S.C., Day A.S. (2018). Exclusive enteral nutrition in children with inflammatory bowel disease: Physician perspectives and practice. JGH Open.

[68] Miele E., Shamir R., Aloi M., Assa A., Braegger C., Bronsky J., de Ridder L., Escher J.C., Hojsak I., Kolaček S. (2018). Nutrition in Pediatric Inflammatory Bowel Disease: A Position Paper on Behalf of the Porto Inflammatory Bowel Disease Group of the European Society of Pediatric Gastroenterology, Hepatology and Nutrition. J. Pediatr. Gastroenterol. Nutr..

